# Chemically Cross-Linked Poly(acrylic-*co*-vinylsulfonic) Acid Hydrogel for the Delivery of Isosorbide Mononitrate

**DOI:** 10.1155/2013/340737

**Published:** 2013-10-23

**Authors:** Talib Hussain, Mahvash Ansari, Nazar Muhammad Ranjha, Ikram Ullah Khan, Yasser Shahzad

**Affiliations:** ^1^Division of Pharmacy and Pharmaceutical Science, University of Huddersfield, Queensgate, Huddersfield HD1 3DH, UK; ^2^Faculty of Pharmacy, Bahauddin Zakariya University, Bosan Road, Multan 68000, Punjab, Pakistan; ^3^College of Pharmacy, GC University Faisalabad, Jinnah Town, Faisalabad 38000, Punjab, Pakistan

## Abstract

We report synthesis, characterization, and drug release attributes of a series of novel pH-sensitive poly(acrylic-*co*-vinylsulfonic) acid hydrogels. These hydrogels were prepared by employing free radical polymerization using ethylene glycol dimethacrylate (EGDMA) and benzyl peroxide (BPO) as cross-linker and initiator, respectively. Effect of acrylic acid (AA), polyvinylsulfonic acid (PVSA), and EGDMA on prepared hydrogels was investigated. All formulations showed higher swelling at high pHs and vice versa. Formulations containing higher content of AA and EGDMA show reduced swelling, but one with higher content of PVSA showed increased swelling. Hydrogel network was characterized by determining structural parameters and loaded with isosorbide mononitrate. FTIR confirmed absence of drug polymer interaction while DSC and TGA demonstrated molecular dispersion of drug in a thermally stable polymeric network. All the hydrogel formulations exhibited a pH dependent release of isosorbide mononitrate which was found to be directly proportional to pH of the medium and PVSA content and inversely proportional to the AA contents. Drug release data were fitted to various kinetics models. Results indicated that release of isosorbide mononitrate from poly(AA-*co*-VSA) hydrogels was non-Fickian and that the mechanism was diffusion-controlled.

## 1. Introduction

Hydrogels are one of the potential polymeric materials that do not dissolve in water at physiological temperature or pH but swell considerably in aqueous media [1]. These are cross-linked polymeric materials in a three-dimensional network which can absorb and retain significant amount of water, making them suitable material for wide range of applications in bioengineering, biomedical, food, and pharmaceutical industries [2, 3]. The water insoluble behavior is attributed to the presence of chemical or physical cross-links which provide the integrity and physical stability to the system. The porous nature of hydrogels facilitates the permeation of water through network structure which is highly influenced by several factors such as chemical composition, hydrophilicity as well as chemical structure of polymer, cross-link densities, and also the functionality of cross-linkers [2].

Stimuli-responsive hydrogels have gained a significant attention and are being developed as drug carrier systems for site specific drug delivery as these hydrogels show dramatic changes in their volume and properties in response to external stimuli such as temperature [4, 5], ionic strength [6], and pH [5, 7, 8]. The optimum use of hydrogel depends upon the properties, namely, equilibrium swelling, swelling kinetics, network permeability, and biocompatibility which can be controlled by adjusting the ratios of monomer to cross-linkers or polymers [9, 10]. Cross-linking is one of the most important factors that affect swelling of the hydrogels. The structure and elasticity of hydrogel are highly dependent on the nature of cross-linking agent as well as on the average molecular mass between the cross-links (*M*_*c*_) [11]. The network structure parameters are critical in describing the mechanical strength, porosity, and releasing mechanics of encapsulated drugs [12, 13]. Previously, pH-sensitive poly(acrylamide-*co*-itaconic acid) hydrogels were synthesized, and influence of network parameters on the swelling and mechanical strength was analyzed. The results clearly demonstrate that network parameters are the key factors in controlling the behavioural changes in the properties of hydrogels [14].

Natural and synthetic materials have been used extensively for the synthesis of hydrogels [15]. Acrylic acid (AA) is deemed to form a super absorbent polymer which can absorb very large amount of water and retain it even under high pressure. As a result of this unique characteristic, it has been used in various controlled drug delivery systems [16, 17]. The swelling behaviour of poly(AA) hydrogels is highly dependent on the pH of the surrounding medium due to the presence of carboxylic groups [18]. Polyvinylsulfonic acid (PVSA; as sodium salt), which is a polyelectrolyte that has negatively charged sulfonate groups, is a blood compatible polymeric material. Due to negatively charged character of sulfonate groups, this polymer may be used as coating material [1].

Here we report AA/PVSA based hydrogels for controlled delivery of a model drug, namely, isosorbide mononitrate. Isosorbide mononitrate is an organic nitrate used in prophylaxis of angina pectoris, acute myocardial infarction, and heart failure [19, 20]. In this respect, a series of hydrogels of AA and PVSA chemically cross-linked using ethylene glycol dimethacrylate (EGDMA) were synthesized via free radical polymerization technique. The prepared hydrogels were subjected to dynamic and equilibrium swelling studies while the drug release was studied in various physiological mimic solutions. Influence of structural parameters on the prepared hydrogels was studied. Any drug-to-polymer interactions were studied using Fourier-transformation infrared spectroscopy (FTIR) while thermal effects were analysed using differential scanning calorimetry (DSC) and thermogravimetric analysis (TGA).

## 2. Experimental

### 2.1. Materials

Isosorbide mononitrate was a gift sample from Hamaz Pharmaceuticals Ltd., Multan, Pakistan. Acrylic acid and vinylsulfonic acid as its sodium salt were sourced from Sigma-Aldrich, UK, and used without further purification. Ethanol, ethylene glycol dimethacrylate (EGDMA), and benzyl peroxide (BPO) were purchased from Merck, Germany, and used as received with a minimum purity of 99%. Double-distilled water was used throughout the experiments.

### 2.2. Synthesis of AA/VSA Polymeric Network

A series of AA/VSA hydrogels were synthesized after the modification of procedure reported earlier [21]. Composition of prepared hydrogels is summarized in Table 1 which were based on the previous experiments. Essentially, weighed amount of VSA (as sodium salt) was dissolved in water in a 50 mL round bottom flask at ambient temperature under constant stirring. Varying amounts of EGDMA and BPO were dissolved in AA in a separate flask. Both mixtures were then mixed thoroughly under continuous stirring until homogenized and the final weight up to 100 g was achieved with double-distilled water. This solution was poured into glass tubes having 16 mm internal diameter and 150 mm length. Each test tube was purged with nitrogen gas for 10–20 minutes to remove air bubbles. These tubes were capped and then placed in water bath maintained at a temperature of 50°C for 48 h. After this period, tubes were taken out and cooled to room temperature. The hydrogels obtained were cut into discs of 6 mm length and immersed into 50 : 50 v/v ethanol-water solution for complete removal of catalyst and unreacted monomers. Gel discs were thoroughly washed until the pH of solution was same as the solution before washing. The hydrogels obtained were dried at 40°C until a constant weight was achieved and then were stored in vacuum desiccators for further use. 

### 2.3. Swelling Studies

The dynamic swelling ratio (*Q*) was evaluated gravimetrically in 100 mL 0.05 M USP phosphate buffer solutions of various pHs, that is, 1.2, 5.5, 6.5, and 7.5. Preweighed dried hydrogels discs were immersed in solutions of desired pH at a temperature of 37°C. Swollen gels were removed from the medium at a predetermined interval of time for 8 hours, weighed after blotting them dry with filter paper, and placed in the same bath. The swelling ratio (*Q*) was calculated from the following [22]:

(1) $$\begin{array}{c} Q=\frac{W_{S}-W_{d}}{W_{d}}, \end{array}$$

where *W*_*s*_ and *W*_*d*_ are the weights of swollen and dried hydrogels, respectively.

### 2.4. Characterization of Poly(AA-*co*-VSA) Hydrogels

#### 2.4.1. Structural Parameters

The average molar mass of the chains between cross-links (*M*_*c*_), directly related to the cross-link density, is an important parameter in characterizing the structural parameters of  hydrogels. Physical and mechanical properties of cross-linked hydrogels are significantly influenced by the *M*_*c*_. According to Flory-Rehners theory [23], *M*_*c*_ can be calculated using

(2) $$\begin{array}{c} M_{c}=\frac{-V_{1}d_{p}\left(V_{S}^{1/3}-V_{S/2}\right)}{\ln\left(1-V_{S}\right)+V_{S}+\chi V_{S}^{2}}, \end{array}$$

where *V*_*S*_ is the volume fraction of the swollen hydrogel at equilibrium, *V*_1_ is the molar volume of the solvent (mL mol^−1^), *χ* is Flory-Huggin's solvent interaction parameter between solvent and polymer [24], and *d*_*p*_ is the density of gel (g mL^−1^).

*χ* can be calculated from

(3) $$\begin{array}{c} \chi =\frac{\ln\left(1-V_{S}\right)+V_{S}}{V_{S}}. \end{array}$$

Volume fraction of the polymer (*V*_*S*_) was calculated by the following equation [25]:

(4) $$\begin{array}{c} V_{S}={\left[1+\frac{d_{p}}{d_{s}}\left(\frac{M_{a}}{M_{b}}-1\right)\right]}^{-1}, \end{array}$$

where *d*_*p*_ and *d*_*s*_  are densities (g mL^−1^) of gel and solvent, respectively. *M*_*a*_ and *M*_*b*_ are the masses (g) of the swollen and dry hydrogels, respectively.

The number of links between two cross-linked chains is called cross-linked density (*q*) which was calculated from [26]

(5) $$\begin{array}{c} q=\frac{d_{p}N_{A}}{M_{C}}, \end{array}$$

where *N*_*A*_ is Avogadro's number (6.023 × 10^23^/mole).

#### 2.4.2. Fourier-Transformation Infrared Spectroscopy (FTIR)

Dried discs of hydrogel samples were powdered in pestle and mortar. The powdered material was mixed with potassium bromide (Merck IR spectroscopy grade) in 1 : 100 proportion and dried at 40°C. The mixture was compressed to semitransparent disc of 12 mm diameter by applying a pressure of 65 kN (pressure gauge, Shimadzu) for 2 minutes. The FTIR spectrum over the wave length range 4000–400 cm^−1^ was recorded using FTIR spectrometer (FTIR 8400 S, Shimadzu).

#### 2.4.3. Thermal Analysis

Differential scanning calorimetry (DSC) and thermogravimetric analysis (TGA) were performed to characterize hydrogel for thermal stability. DSC was done in the DSC unit (Netzsch DSC-200 PC Phox, Germany). The samples were heated in a close aluminum pan at the rate of 40°C/min under nitrogen flow (50 mL min^−1^). TGA was performed using a thermogravimetric analyzer (TGA 951, TA Instruments, USA). Samples were heated at the rate of 10°C/min with temperature range of 30–400°C.

#### 2.4.4. Drug Loading and Release Kinetics

Dried hydrogel discs were immersed in 10 mL of isosorbide mononitrate solution (1% w/v) prepared in ethanol-water mixture (50 : 50% v/v). These hydrogels were kept at ambient temperature without stirring for 7 days to attain equilibrium swelling. After reaching equilibrium swelling point, discs were removed from the loading solution, blotted with filter paper, and dried in an oven at 45°C for 7 days. The amounts of drug loaded were calculated by recurrently extracting the drug from the hydrogels in ethanol-water mixture (50 : 50% v/v) and the concentration of drug in pooled extract was monitored spectrophotometrically at 210 nm. The experiments were conducted in triplicate.

Drug release studies were carried out in USP II dissolution apparatus (Pharma Test, Germany) using 0.05 M USP phosphate buffer solutions at various physiological pHs (1.2, 6.5, and 7.5). The weighed hydrogel discs were immersed in 500 mL dissolution medium stirred at a rate of 100 rpm, and maintained at 50°C. With each sampling, 5 mL release media was withdrawn at predetermined time and immediately replenished with the same volume of fresh medium to maintain sink conditions. The determination of isosorbide mononitrate release was carried out at 210 nm for up to 12 h at regular intervals. Drug release data were fitted to various kinetic models including zero-order, first-order, Higuchi, and Korsmeyer-Peppas models [27, 28]. These models are generally used when more than one type of release phenomena is involved.

## 3. Results and Discussion

### 3.1. Swelling Behaviour

**Figure 1:**
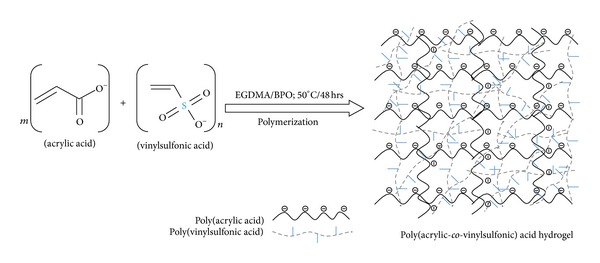
Schematic representation of hydrogel synthesis.

The poly(acrylic*-co-*vinylsulfonic) acid hydrogels were synthesized using AA as the monomer, PVSA as the polymer, and EGDMA as the cross-linker in the presence of BPO as an initiator of free radical polymerization. Schematic depiction of the synthesis of cross-linked hydrogel is shown in Figure 1. Since AA and VSA are negatively charged polyelectrolytes in the polymerization medium; therefore, strong electrostatic repulsive forces would operate between AA (COO^−^) and VSA (SO_3_^−^) groups. It is envisaged that a possible expanded network of poly(AA/VSA) hydrogel would be obtained that has a higher swelling capacity [29]. 

Swelling behaviour of hydrogels against external stimuli (temperature or pH) is a measure of their usefulness as biomaterials in the field of pharmaceuticals. Mostly, ionic hydrogels are sensitive to pH and swell significantly at a pH that favours their swelling. In the current study, swelling behaviour of the synthesized hydrogels in 0.05 M USP phosphate buffer solution of various pHs (1.2, 5.5, 6.5, and 7.5) was determined gravimetrically. Two factors, namely, contents (AA, PVSA, and EGDMA) and pH of the buffer, were considered in swelling behaviour studies. It is well established that negatively charged groups tend to repel each other through electrostatic repulsion; thus, a high swelling ratio was anticipated for the hydrogels. 

**Figure 2:**
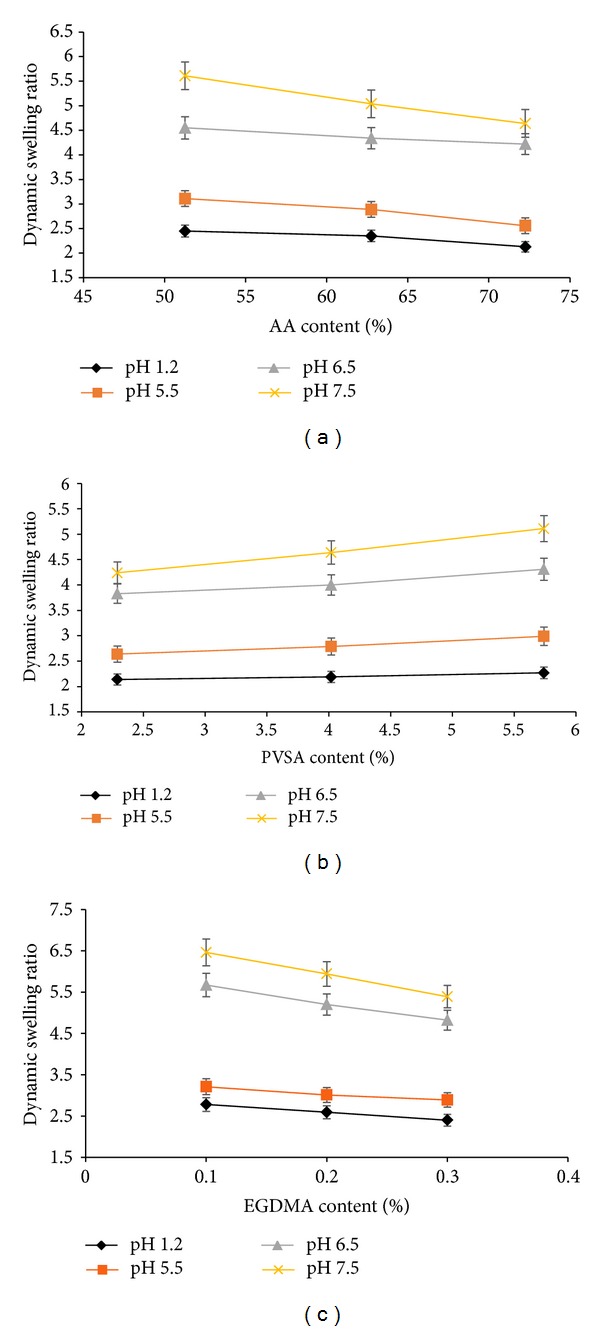
Dynamic swelling at various pHs as a function of AA (a), PVSA (b), and EGDMA contents (c); error bars indicate SD (*n* = 3).

As can be seen from Figures 2(a)–2(c), swelling behaviour was directly related to the concentration of AA, PVSA, and EGDMA. In this study, decrease in swelling behaviour was observed at all pHs with the increase in AA (S_1_, S_2_, and  S_3_) and EGDMA (S_7_, S_8_, and  S_9_) content while an increased swelling ratio was achieved with increasing concentration of PVSA (S_4_, S_5_, and  S_6_). This could be attributed to the fact that AA is a small molecule and increasing the content causes smaller nodes with increase in cross-linking and decrease of cross-link density. So these factors contribute to less swelling with increasing concentrations of AA and EGDMA. This is further confirmed by studies on structural parameters of hydrogels (see Section 2.2). Secondly, increased AA content may influence the preference of homopolymerization over copolymerization; thus, swelling ratio decreased at higher AA content [30, 31]. On the other hand, increasing the PVSA content of the polymer network was found to increase the swelling of the hydrogels. This might be explained on the basis that PVSA is more hydrophilic than AA and has more swelling capability. Since VSA has negatively charged sulfonic groups, the interchain repulsion could be another cause of the expansion of the polymeric network which leads to the higher swelling ratio [1].

Buffer pH was the second factor in the swelling studies of poly(AA*-co-*VSA) hydrogels. Dynamic swelling ratio was increased with the change of buffer pH from acidic to alkaline. Alkaline pH promotes enhanced ionization of carboxylic and sulfonic groups; therefore, inter-chain ionic repulsion increases the swelling capacity. Also, an increase in the anion density within the hydrogel can enhance the osmotic pressure inside the gel network. This difference in osmotic pressure between the internal and external gel is balanced by the swelling of the hydrogel.

### 3.2. Characterization of Hydrogels

The suitability of a hydrogel as a potential drug delivery system is dependent on its bulk structure. Most importantly, network parameters of a hydrogel are directly related to the swelling and have an influence on the release of the drug from the hydrogels. In this study, therefore, we have characterized network structure in terms of average molar mass of the chains between two consecutive cross-links (*M*_*c*_), volume fraction of swollen gel (*V*_*S*_), cross-link density (*q*), and Flory-Huggins solvent interaction parameter between solvent-polymer interactions (*χ*), and the results are presented in Table 2. It was found that, with increase in AA and EGDMA content, the cross-link density (*q*) increases, causing a decrease in the average mass between the two cross-links (*M*_*c*_). This results in decreased free volume available; thus an insufficient space is available for the water molecules to diffuse into the hydrogel; consequently, a decreased swelling was achieved. However, increasing PVSA content resulted in a decreased cross-link density (*q*), causing an increase in the mass between the two neighbouring cross-links (*M*_*c*_) which provides sufficient space for the water molecules to diffuse into the hydrogel and a higher swelling was achieved.

The polymer volume fraction (*V*_*s*_), calculated using (5), ranged from 0.16 to 0.22 for S_1_, S_2_, and  S_3_; from 0.14 to 0.24 for S_4_, S_5_, and  S_6_; and from 0.14 to 0.16 for S_7_, S_8_, and  S_9_, respectively. The volume fraction (*V*_*s*_) of the polymer in the swollen state describes the amount of water that can be imbibed into a hydrogel and is described as the ratio of the polymer volume to the swollen gel [12]. The volume fraction of polymer was increased with an increase in AA and EGDMA content, which, in turn, indicates a decrease in swollen polymer content. Furthermore, this indicates that the distance between two cross-linking points decreases with increasing the content of AA and EGDMA; thus, free volume available decreases. Reverse was true for the formulations in which PVSA content was increased. 

The solvent-polymer interaction (*χ*) was in the range of 0.5 for all formulations indicating that there is a weak interaction between polymer and water and a strong interaction between the polymer chains [32].

**Figure 3:**
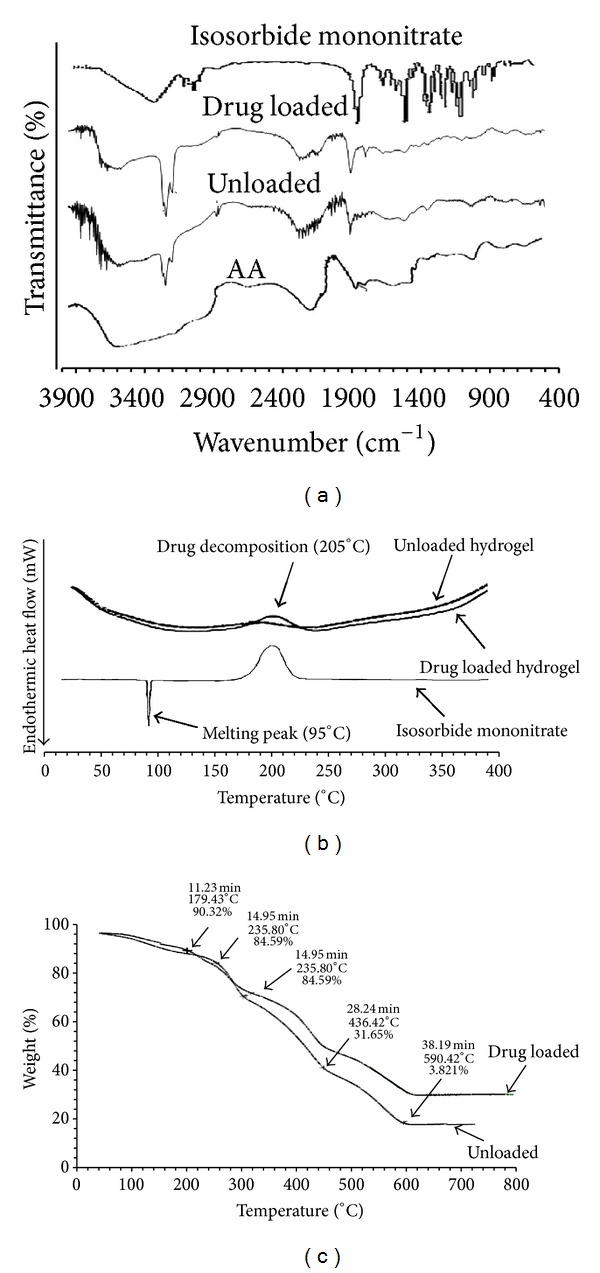
Characterization of hydrogels using (a) FTIR, (b) DSC, and (c) TGA.

In order to confirm interaction between AA and PVSA, samples were analysed using FTIR (Figure 3(a)). The FTIR of AA showed a strong absorption band at 1707 cm^−1^ which is typical of carbonyl (−C=O) stretching of COOH groups of AA. Absorption bands at 1580 cm^−1^, 1458 cm^−1^, and 1401 cm^−1^ are also arisen from AA and assigned to asymmetric −COO stretching vibration, bending vibration of –CH, and symmetric −COO stretching vibration [33]. Peaks at 2954.45 cm^−1^, 2930.18 cm^−1^, and 2854.45 cm^−1^ indicate −OH stretching vibration of carboxylic acid [34]. The FTIR spectra of AA/PVSA hydrogel showed the absorption bands at 1170 cm^−1^ and 1040 cm^−1^  which are due to −SO_3_^−^ and S=O vibration [35]. FTIR spectra indicated the main changes in the region of 2300–3000 cm^−1^  which is evidence of interaction between monomers. It could also be due to the bonds overlapping. From the FTIR it is clear that there is no significant shift in major peaks, which indicates that there is no chemical interaction between the polymer and the drug used.

DSC thermograms of pure drug, unloaded, and drug-loaded hydrogels are presented in Figure 3(b). The thermogram of DSC clearly indicates a sharp melting peak of isosorbide mononitrate at about 95°C followed by a decomposition peak at about 200°C. The drug-loaded hydrogel showed an absence of drug melting peak which indicates molecular dispersion of drug in the prepared hydrogels. However, drug decomposition peak appeared at about 205°C in the drug-loaded hydrogel. The unloaded sample did not show any endothermic transitions due to rigid polymer network structure because of chain entanglement.

TGA thermograms of drug-loaded and unloaded hydrogels are presented in Figure 3(c). Polymer is stable at 100°C; however above 235.80°C there is a substantial loss in copolymer weight. Thermal degradation occurred in two stages. Initially, weight loss of up to 10% is observed in the range of 100–200°C while maximum weight loss is observed at 586.42°C with only 3.8% of polymer left. The temperature range during which specific % age of polymer is degraded is nearly the same for drug-loaded and unloaded hydrogels.

### 3.3. Release Kinetics

The essential condition for the solute to be released from the hydrogel is the existence of a partition phenomenon based on lipophilicity [36], which considers that the partitioning of solute occurs between a solvent and hydrogel phase depending on the partition activity of the solute which expresses the physicochemical affinity of solute for both phases [37]. 

**Figure 4:**
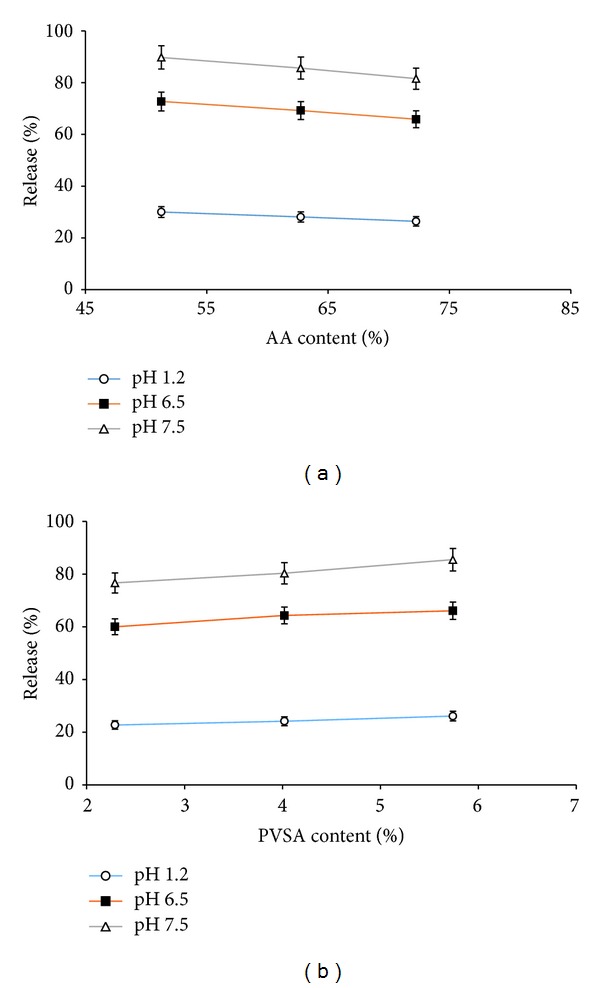
Drug release forms poly(AA*-co-*VSA) hydrogels; error bars indicate SD (*n* = 3).

In the present study, the formulated hydrogels were evaluated for the release of a model drug, namely, isosorbide mononitrate, in USP phosphate buffer solutions of different pHs (1.2, 6.5, and 7.5) at 37°C. It should be noted that only those formulations were selected for release studies which have fixed concentrations of EGDMA (S_1_to  S_6_). The amount of drug loaded in the selected hydrogels is shown in Table 1. Isosorbide mononitrate release profile as a function of AA and PVSA content at various pHs is shown in Figure 4. A decrease in drug release is observed with increase in AA content while drug release was enhanced with increasing the PVSA content in the hydrogels. The release rate of drugs was found to be correlated with the swelling response of the hydrogels against varying pHs of the dissolution media. It can be seen from Figure 4 that the drug release increased by increasing the pH of the medium. As the pH of the medium increases, water uptake by the hydrogels increases which resulted in increased osmotic pressure inside the gel; hence, drug release was enhanced from the swollen gels.

Drug release data were fitted to various kinetics models including zero-order, first-order, Higuchi, and Korsmeyer-Peppas models. Regression coefficient (*R* ^2^) values were obtained for poly(AA*-co-*VSA) hydrogels at varying contents of AA and VSA (Table 3) which indicate that drug release follow Higuchi's model. The *R* ^2^ values showed the highest linearity which means that drug release mechanism was diffusion-controlled [38].

The equation of *M*_*t*_/*M*_*∞*_ = *kt* ^*n*^ was used to study the mechanism of drug release [28], where *M*_*t*_/*M*_*∞*_ is the drug release fraction at time *t*, *k* is the kinetic constant, and *n* is the release exponent describing the release mechanism. When *n* = 0.5, Fickian diffusion, also known as Case I diffusion, occurs; if *n* = 1, drug release follows zero-order or Case II transport; 1 > *n* > 0.5, non-Fickian or anomalous drug transport when the rate of diffusion and polymer relaxation simultaneously occur. It can be seen that the values of release exponent (*n*) were between 0.5 and 1; therefore, drug release from the hydrogel followed non-Fickian release mechanism (Table 3). 

## 4. Conclusion

In the present work pH-sensitive AA/VSA hydrogels have been successfully prepared by free radical polymerization using EGDMA as cross-linking agent and benzyl peroxide as reaction initiator. Hydrogels showed a pH dependent swelling behaviour which was found to be maximum at pH 7.5 of the medium. It was also observed that swelling of hydrogel increases when increasing the concentration of PVSA and decreases when increasing the concentrations of AA and EGDMA in the gels. Structural parameters confirmed different architectural aspects of hydrogels necessary for proper functioning. These gels were successfully loaded with model drug (isosorbide mononitrate) and FTIR spectra confirmed absence of drug polymer interaction. Thermal analysis showed thermal stability of polymeric network and molecular dispersion of drug in the hydrogels. It was observed that drug release decreases when increasing the amount of AA, but it increases when increasing the concentration of PVSA and pH of the medium. Release kinetic analysis revealed a non-Fickian diffusion mechanism for the release of the isosorbide mononitrate. These results suggest that poly(acrylic*-co-*vinylsulfonic) acid hydrogels can be used as potential pH-sensitive drug delivery systems.

## Figures and Tables

**Table 1:** Different formulations of AA/PVSA hydrogels.

Sample	PVSA content (g/100 g solution)	AA content (g/100 g solution)	AA/PVSA (Wt%)	EGDMA content (Wt%)	Drug loading (g/g of hydrogel)
S_1_	6.02	51.26	89.20/10.80	0.2	0.166 ± 0.012
S_2_	6.02	62.75	91.00/09.00	0.2	0.164 ± 0.034
S_3_	6.02	74.25	92.28/07.72	0.2	0.153 ± 0.019
S_4_	2.29	65.52	96.61/03.39	0.2	0.116 ± 0.028
S_5_	4.02	65.52	94.21/05.79	0.2	0.144 ± 0.043
S_6_	5.74	65.52	91.93/08.07	0.2	0.159 ± 0.017
S_7_	6.97	62.06	90.00/10.00	0.1	nd*
S_8_	6.97	62.06	90.00/10.00	0.2	nd*
S_9_	6.97	62.06	90.00/10.00	0.3	nd*

*nd corresponds to not determine.

**Table 2:** Structural parameters of hydrogels.

Sample	*M*_*c*_ (g mol^−1^)	*q*	*V*_*s*_	*χ*
S_1_	379.08	5.71	0.16	0.56
S_2_	279.61	7.19	0.19	0.57
S_3_	219.97	9.62	0.22	0.58
S_4_	184.66	7.29	0.24	0.59
S_5_	210.58	5.54	0.23	0.59
S_6_	281.91	4.95	0.12	0.57
S_7_	504.54	9.62	0.14	0.55
S_8_	422.05	10.77	0.15	0.55
S_9_	378.48	12.92	0.16	0.56

**Table 3:** Drug release kinetics of poly(AA*-co-*VSA) hydrogels in solutions of different pHs.

Sample	pH	Zero-order kinetics		First-order kinetics		Higuchi model		Korsmeyer-Peppas model		
		*K*_0_ (h^−1^)	*R* ^2^	*K*_1_ (h^−1^)	*R* ^2^	*K*_2_ (h^−1^)	*R* ^2^	*n*	*R* ^2^	Release mechanism
S_1_	1.2	2.4370	0.9861	0.0292	0.9925	0.1039	0.9838	0.8399	0.9939	Non-Fickian
	5.5	6.0195	0.9675	0.1081	0.9982	0.2605	0.9952	0.9051	0.9865	Non-Fickian
	7.5	7.2830	0.9495	0.1840	0.9914	0.3181	0.9952	0.8515	0.9797	Non-Fickian
S_2_	1.2	2.2615	0.9848	0.0268	0.9931	0.0969	0.9928	0.8150	0.9977	Non-Fickian
	5.5	5.7970	0.9840	0.0982	0.9980	0.2482	0.9908	0.9231	0.9950	Non-Fickian
	7.5	6.8438	0.9750	0.1504	0.9766	0.2951	0.9961	0.8691	0.9892	Non-Fickian
S_3_	1.2	1.9763	0.9662	0.0232	0.9766	0.0855	0.9927	0.7735	0.9884	Non-Fickian
	5.5	5.3641	0.9385	0.0894	0.9866	0.2353	0.9917	0.9122	0.9667	Non-Fickian
	7.5	6.4905	0.9375	0.1383	0.9968	0.2851	0.9939	0.8438	0.9662	Non-Fickian
S_4_	1.2	1.8342	0.9438	0.0212	0.9570	0.0802	0.9914	0.7869	0.9840	Non-Fickian
	5.5	4.8496	0.9626	0.0749	0.9929	0.2106	0.9969	0.9211	0.9746	Non-Fickian
	7.5	6.3101	0.9885	0.1181	0.9888	0.2695	0.9907	0.9147	0.9947	Non-Fickian
S_5_	1.2	1.8812	0.9704	0.0219	0.9813	0.0184	0.9980	0.7669	0.9876	Non-Fickian
	5.5	5.4079	0.9664	0.0874	0.9964	0.2340	0.9939	0.9493	0.9846	Non-Fickian
	7.5	6.4510	0.9735	0.1308	0.9934	0.2786	0.9977	0.8571	0.9864	Non-Fickian
S_6_	1.2	2.1164	0.9762	0.0249	0.9856	0.0910	0.9923	0.8174	0.9921	Non-Fickian
	5.5	5.2759	0.9515	0.0884	0.9923	0.2304	0.9965	0.8545	0.9754	Non-Fickian
	7.5	6.4930	0.9748	0.1348	0.9816	0.2800	0.9961	0.8392	0.9898	Non-Fickian
